# Results of urinary bacterial cultures and antibiotic susceptibility testing of dogs and cats in the UK

**DOI:** 10.1111/jsap.13406

**Published:** 2021-08-31

**Authors:** J. D. Fonseca, D. E. Mavrides, P. A. Graham, T. D. McHugh

**Affiliations:** ^1^ Centre for Clinical Microbiology University College London Royal Free Campus London NW3 2QG UK; ^2^ Department of Veterinary Medicine University of Cambridge Madingley Road Cambridge CB3 0ES UK; ^3^ School of Veterinary Medicine and Science University of Nottingham Sutton Bonington LE12 5RD UK

## Abstract

**Objectives:**

Bacterial urinary tract infections are a common diagnosis in small animal practice and antibiotics are often administered empirically. The aim of this study was to investigate the aetiology and antibiotic resistance of uropathogens in dogs and cats in the UK.

**Materials and Methods:**

Retrospective study of uroculture and antibiotic susceptibility testing results (n=808) by disk diffusion processed at a veterinary pathology laboratory between 2011 and 2012.

**Results:**

Significant bacteriuria was detected in 18.4% of samples from dogs and 10.0% from cats, most of which (>90%) yielded a single organism. *Escherichia coli* was the most prevalent bacterial species (54.7% and 55.6% of feline and canine isolates, respectively) followed by *Proteus mirabilis* in dog samples (22.7%) and *Enterococcus* spp. in cat samples (23.2%). Approximately a third of *E. coli* isolates were resistant to ampicillin but resistance was much lower among *Enterococcus* spp. and *P. mirabilis*. Resistance to amoxicillin‐clavulanic acid also seemed to be emerging, particularly in *E. coli* (almost 20% resistant). In contrast, resistance to trimethoprim‐sulfamethoxazole for uropathogens remained <13% except for *P. mirabilis* (19.4%). Overall, fluoroquinolones showed the best in vitro activity (resistance mostly below 10% for enrofloxacin and marbofloxacin).

**Clinical Significance:**

Our results provide evidence of the emergence of resistance to antibiotics commonly used to treat bacterial urinary tract infections. Continued monitoring of the patterns of antibiotic resistance in uropathogens is needed to assess the adequacy of recommendations on the empiric therapy of these infections.

## INTRODUCTION

Bacterial urinary tract infections (UTIs) are diagnosed in approximately 14% of dogs during their lifetime (Ling 1984). UTIs are more common in cats aged 10 or above, those with concurrent diseases (*e.g*. diabetes mellitus, chronic renal failure) and those that have undergone urinary catheterisation or perineal urethrostomy (Lulich *et al*. 1992, Litster *et al*. 2007). UTIs can cause a spectrum of disease from asymptomatic to life threatening. The clinical signs known to be compatible with UTI include dysuria, stranguria, pollakiuria and hematuria, but such signs are not pathognomonic of infection. A diagnosis of UTI can be aided by the detection of significant bacteriuria in quantitative cultures of urine specimens (Weese *et al*. 2011, Olin & Bartges 2015). In the same manner, resolution of clinical signs is not a sign of infection elimination and cure (especially in chronic or recurrent UTIs) and can only be determined with urine culture, ideally 5 to 7 days after therapy is terminated (Barsanti & Finco 1979, Ball *et al*. 2008, Olin & Bartges 2015).

Urine samples for microbiological examination should ideally be collected from all patients suspected of bacterial UTI before the initiation of antibiotic treatment to confirm the diagnosis and determine the aetiology of the infection. Empirical treatment, while laboratory results are pending, is usually recommended to relieve patient discomfort (Weese *et al*. 2011). An aetiological diagnosis may not be pursued due to financial constraints of the pet owner and/or a good initial response to empirical antibiotics. Consequently, continuation with the selected treatment occurs without any testing. Current knowledge on the local susceptibility patterns of the most common uropathogens is therefore necessary to ensure adequate empiric antibiotic coverage. Antibiotic choices should also take into account the therapeutic levels reached in urine; therefore antibiotics with high renal excretion rates are preferred (Kramer *et al*. 2012). Inadequate empirical choices can lead to poor patient outcomes and contribute to the selection of resistance.

The emergence of multidrug‐resistant (MDR) UTIs, which has already been reported (Cohn *et al*. 2003, Penna *et al*. 2010), can seriously complicate the treatment of these infections by limiting the effective drugs available. There are also public health concerns associated with the potential zoonotic transmission of some of these resistant organisms, which is facilitated by the close proximity between humans and their household pets (Starcic *et al*. 2002, Guardabassi *et al*. 2004, Weese 2008, Rampacci *et al*. 2018).

The objectives of the present study were to provide evidence on the aetiology and antibiotic resistance of bacterial uropathogens in dogs and cats in the UK using retrospective data from referrals to a diagnostic laboratory.

## MATERIALS AND METHODS

### Data collection

**FIG 1:**
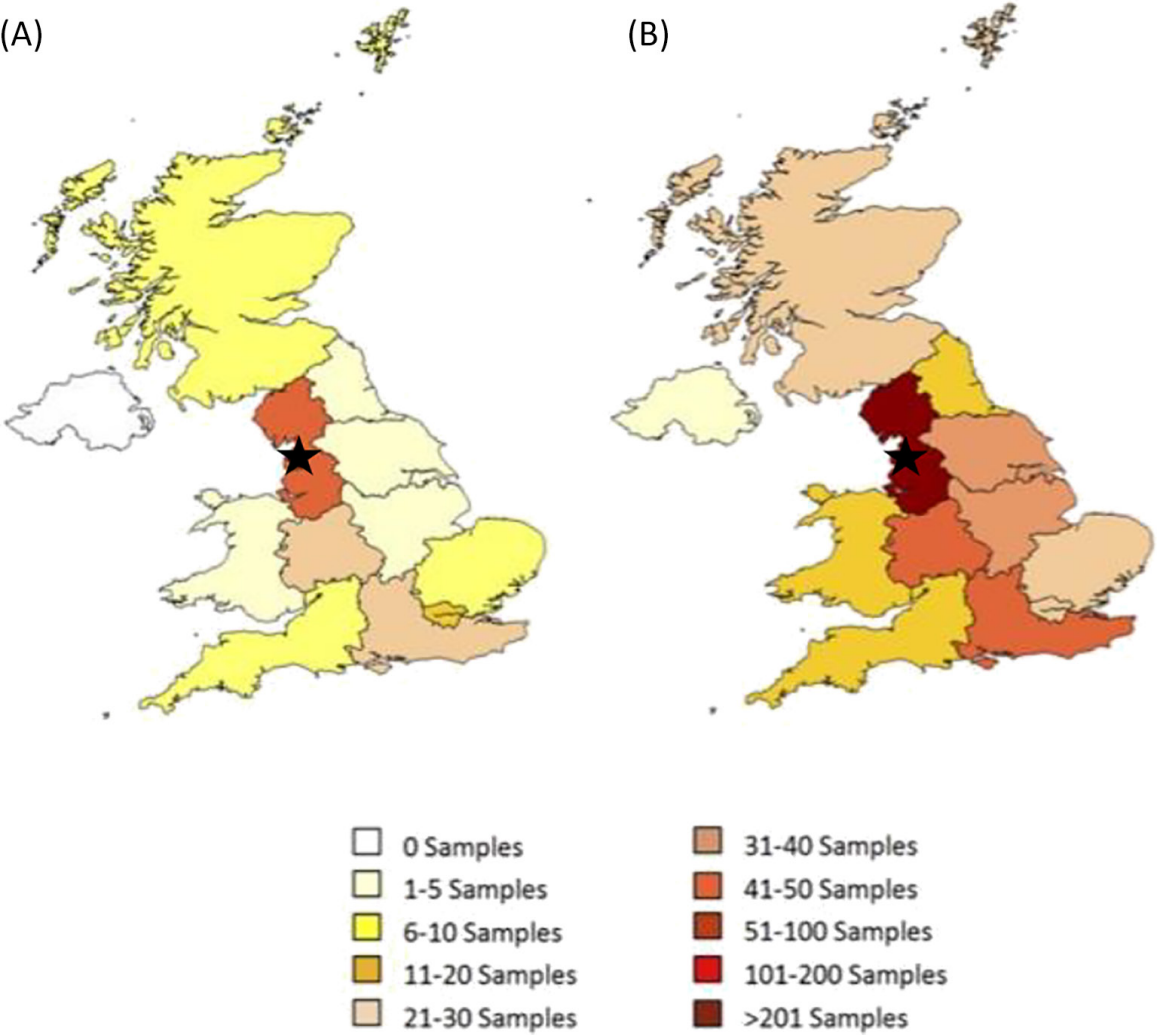
Geographic distribution of veterinary practices by number of (A) feline and (B) canine samples with significant bacteriuria (cut‐off of ≥105 colony‐forming units (CFU)/mL and growth of up to two possible uropathogens). ★Location of NationWide Laboratories

Data from urine samples from dogs and cats with significant bacteraemia submitted to NationWide Laboratories (NWL; Lancashire, UK) for microbiological examination between November 2011 and November 2012 were retrieved from electronic records. The geographic distribution of the veterinary practices from which the samples were originated is shown in Fig 1. Information on the method of urine collection was not provided by the requesting veterinarian. Urine samples of an animal were only submitted once (non‐repetitive samples). The collected data included age and gender of the patients and the results from uroculture and antibiotic susceptibility testing. A cut‐off of ≥10^5^ colony‐forming units (CFU)/mL and growth of up to two possible uropathogens was used to define clinically significant bacteriuria (Sørensen *et al*. 2016). Only samples that matched these criteria were included in the analysis and if more than two isolates were cultured this was considered contaminated. Information on the clinical status of the patient (signs of a UTI or subclinical infections) was not provided but samples were submitted for clinical purposes, *e.g*. clinical suspicion of a UTI or screening susceptible patients.

### Laboratory methods

Semi‐quantitative bacterial cultures of undiluted urine samples were performed by the calibrated loop method. A 10‐μL loop was used to inoculate the urine onto 5% blood agar and MacConkey agar. The plates were placed at 37°C and checked for growth after 18 to 24 hours of incubation. Bacteria were identified by Gram stain and standard biochemical procedures. Antimicrobial susceptibility testing was done by the Kirby‐Bauer method according to the recommendations of the Clinical and Laboratory Standards Institute (CLSI 2008). Quality control strains (*Staphylococcus aureus* ATCC 25923, *Escherichia coli* ATCC 25922, *Pseudomonas aeruginosa* ATCC 27853) were prepared and tested by exactly the same methods as employed for the test isolates. NWL where the microbiological testing was carried out held an ISO17025 accreditation for clinical microbiology testing of urine. The antibiotics tested were penicillin, ampicillin, amoxicillin‐clavulanic acid, cephalothin, cefovecin, tetracycline, enrofloxacin, marbofloxacin, erythromycin and trimethoprim‐sulfamethoxazole. Clinical breakpoints defined by CLSI (CLSI 2008) were applied and noted differences in breakpoint data between CLSI (2008) and CLSI VET01S (2020) are included in Table 1.

**Table 1:** Clinical breakpoints used were from CLSI (2008) except for cefovecin which were obtained from Zoetis’ breakpoint data. The right part of the table indicates the breakpoint changes for amoxicillin‐clavulanic acid and tetracycline (CLSI 2020)

Antimicrobial agent	Disk content	CLSI (2008)				CLSI VET01S (2020)			
		Zone diameter (mm)				Zone diameter (mm)			
		S	I	R	Bacterial species	Disk content	S	I	R
Penicillin (human)									
Staphylococci	10 units	≥29	–	≤28					
Enterococci	10 units	≥15	–	≤14					
Ampicillin (human)									
Enterobacteriales	10 μg	≥17	14 to 16	≤13					
Staphylococci	10 μg	≥29	–	≤28					
Enterococci	10 μg	≥17	–	≤16					
Amoxicillin‐clavulanic acid (human)									
Staphylococci	20/10 μg	≥20	–	≤19					
Other organisms	20/10 μg	≥18	14 to 17	≤13	*E. coli* (dogs and cats)	20/10 μg	≥18	–	–
Cephalothin (human)	30 μg	≥18	15 to 17	≤14					
Cefovecin (dogs/cats)									
Enterobacteriales/non‐enterobacteriales	30 μg	≥24		≤20					
Staphylococci	30 μg	≥24		≤20					
Tetracycline (human)									
Organisms other than streptococci (includes Enterobacteriales and *Staphylococcus* spp.)	30 μg	≥19	15 to 18	≤14	Enterobacteriales (human)	30 μg	≥15	12 to 14	≤11
Enrofloxacin									
Dogs	5 μg	≥23	17 to 22	≤16					
Marbofloxacin									
Dogs	5 μg	≥20	15 to 19	≤14					
Erythromycin (human)									
*Enterococcus* spp.	15 μg	≥23	14 to 22	≤13					
*Staphylococcus* spp.	15 μg	≥23	14 to 22	≤13					
Trimethoprim‐sulfamethoxazole (human)									
*Staphylococcus* spp.	1.25/23.75 μg	≥16	11 to 15	≤10					
Enterobacteriales	1.25/23.75 μg	≥16	11 to 15	≤10					

S Sensitive, I Intermediate, R Resistant

Breakpoints for dogs have been extrapolated to cats

### Data analysis

Data records were exported in a single batch from the Laboratory Information system by NWL (Winpath, Clinisys) using the application's query function. All records with a species code “canine” or “feline” and a test request code “urine culture” were included in a comma‐separated format that was then imported into Microsoft Excel. Results were further analysed by a single operator. Results were expressed as percentages. The frequency of different bacterial species in urine specimens was calculated by dividing the number of isolates from a certain species by the total number of isolates of this species from the canine and feline samples. For each antibiotic, the percentage of resistance was calculated by dividing the number of resistant isolates by the total number of isolates reported.

## RESULTS

Microbiological results were available for a total of 5170 non‐repetitive urine samples, 3460 from dogs and 1710 from cats. Bacterial growth was recorded for 1547 of these samples but only 808 [637 (18.4%) from dogs and 171 (10.0%) from cats] matched our inclusion criteria. Significant bacteriuria was detected in 18.4% of samples from dogs and 10.0% from cats, most of which (>90%) yielded a single organism.

### Age and sex of the patients

Information about the age and gender of the patient was available for 152 of 171 samples from cats and 586 of 637 samples from dogs. Age and gender distributions are shown in Table 2. More than half of the samples from dogs and cats (808) belonged to female dogs (52.7%; n=426); for cats this figure amounted to 10.4% (n=84).

**Table 2:** Age and sex distribution of canine and feline patients with significant bacteriuria

	Number of samples (%)					Total dogs
	Cats			Dogs		
	Males	Females	Total cats	Males	Females	
Age groups (years)						
<1	2 (2.9)	0 (0)	2 (1.3)	11 (6.9)	49 (11.5)	60 (10.2)
1–4	14 (20.6)	9 (10.7)	23 (15.1)	27 (16.9)	73 (17.1)	100 (17.1)
5–7	8 (11.8)	9 (10.7)	17 (11.2)	26 (16.3)	86 (20.2)	112 (19.1)
8–10	13 (19.1)	16 (19.0)	29 (19.1)	47 (29.4)	116 (27.2)	163 (27.8)
≥11	31 (45.6)	50 (59.5)	81 (53.3)	49 (30.6)	102 (23.9)	151 (25.8)
Age range	2 months to 20 years	1–31 years		3 months to 16 years	2 months to 17 years	

Percentages in brackets were calculated by dividing the number of samples in each age group by the total number of samples from each gender and multiplying by 100

### Bacterial isolates

Most urine specimens from both cats (94.2%) and dogs (94.3%) yielded the pure growth of a single organism. Two different isolates were present in the remaining samples (approximately 6% for cat and dog samples each).

Gram‐negative species predominated, particularly those belonging to the Enterobacteriales which represented 57.3% and 76.6% of the isolates obtained from feline and canine samples, respectively. *E. coli* was the most frequently isolated bacterium; 54.7% in cats and 55.6% in dogs (Table 3), followed by *Proteus mirabilis* in dog samples and *Enterococcus* spp. in cat samples. Additionally, almost 10% of isolates from cats yielded coagulase‐negative staphylococci. SIG was the most frequently isolated coagulase‐positive *Staphylococcus* with 4.4% and 7% of isolates from cat and dog samples, respectively.

**Table 3:** Bacterial species isolated from urine samples with significant bacteriuria

Bacterial species	Percentage of isolates (%)	
	Cats (n=171)	Dogs (n=637)
Gram‐negative	59.7 (102)	79.0 (503)
Enterobacteriales	57.3 (98)	76.6 (488)
*Escherichia coli*	54.7 (94)	55.6 (354)
*Proteus mirabilis*	2.2 (4)	22.7 (145)
Gram‐positive	40.3 (69)	21 (134)
*Enterococcus* spp.	23.2 (40)	6.3 (40)
β‐haemolytic streptococci	1.7 (3)	5.6 (36)
Coagulase‐negative staphylococci	9.9 (17)	2.3 (15)
*Staphylococcus intermedius* group	4.4 (8)	7.0 (45)

The most common combinations in canine samples that yielded mixed bacterial growth were *E. coli*/β‐haemolytic streptococci (n=8), *E. coli*/*P. mirabilis* (n=7), *P. mirabilis*/*Enterococcus* spp. (n=5), *E. coli*/*Enterococcus* spp. (n=4) and *Staphylococcus intermedius* Group/β‐haemolytic streptococci (n=4). *E. coli*/*Enterococcus* spp. (n=6) was the most common combination in feline samples.

### Antibiotic susceptibility

The great majority of isolates obtained from cat and dog samples (96.7% and 96.1%, respectively) were susceptible to at least one of the oral antibiotics commonly prescribed to UTI patients (ampicillin, amoxicillin‐clavulanic acid, cephalothin, tetracycline and trimethoprim‐sulfamethoxazole). Out of these, trimethoprim‐sulfamethoxazole and amoxicillin‐clavulanic acid were the drugs with the lowest percentage of resistance (Table 4). Resistance above 20% was detected to cephalothin and cefovecin for *E. coli*, with the highest resistance against cephalothin (64.4%). Overall, 36.9% of *E. coli* isolates from canine samples and 39.4% from feline samples were resistant to ampicillin and almost 20% were resistant to amoxicillin‐clavulanic acid in cats and dogs. Resistance to erythromycin and tetracycline was detected in approximately 90% and 50% of enterococcal isolates respectively, and 10% of the canine enterococci were resistant to ampicillin and amoxicillin‐clavulanic acid, whereas no resistance was observed among the feline enterococci. Fluoroquinolones showed good in vitro activity against the isolates included in our study (resistance 0–7.3% for enrofloxacin and marbofloxacin) excluding coagulase‐negative staphylococci with 0% to 17.6% resistance. Staphylococci isolates displayed high resistance to penicillin and ampicillin, but resistance to trimethoprim‐sulfamethoxazole and cefovecin was much lower (Table 4).

**Table 4:** Antibiotic resistance profiles of bacteria isolated from urine samples with significant bacteriuria

Bacterial species	Animal species	Antibiotic (% of resistant isolates)									
		P*	AMP*	AMC*	CEF*	CVN†	TET‡	ENR†	MAR†	E‡	SXT*
*Escherichia coli*	Dogs		36.9	16.8	64.4	31.2	11.1	7.3	6.5		12.6
	Cats		39.4	19.2	58.8	26.8	19.2	7.1	7.1		8.2
*Proteus mirabilis*	Dogs		11.7	4.8	6.9	2.8		4.1	0.7		19.4
	Cats§		–	–	–	–		–	–		–
*Enterococcus* spp.	Dogs	10	10	10			47.5			90	
	Cats	0	0	0			50			96.7	
*Staphylococcus intermedius* Group	Dogs	80	80			0	24.4	4.4	0		11.1
	Cats§	50	50			0	12.5	0	0		0
Coagulase‐negative staphylococci	Dogs	60	60			20	40	13.3	13.3		6.7
	Cats	17.6	17.6			11.8	5.9	17.6	0		11.8

P Penicillin, AMP Ampicillin, AMC Amoxicillin‐clavulanic acid, CEF Cephalothin, CVN Cefovecin, TET Tetracycline, ENR Enrofloxacin, MAR Marbofloxacin, *E* Erythromycin, SXT Trimethoprim‐sulfamethoxazole

Inferred resistance for certain antibiotics: Amoxicillin inferred from ampicillin, first‐generation cephalosporins (*e.g*. cephalexin) inferred from cephalothin, doxycycline inferred from tetracycline

^*^ Antibiotics commonly used in the treatment of uncomplicated UTIs

^†^ Antibiotics used second line for the treatment of UTIs, only where there is culture and sensitivity evidence that first‐line drugs will not be effective

^‡^ Antibiotics not recommended for the treatment of UTIs

^§^ n < 10

## DISCUSSION

Inadequate empirical choices or antibiotic treatment of nonbacterial conditions such as feline idiopathic/interstitial cystitis can lead to poor patient outcomes and contribute to the selection of resistance, particularly given the long courses of antibiotic treatment usually preferred in companion animal medicine compared to human treatment regimes (Rampacci *et al*. 2018). Previous veterinary guidelines (Weese *et al*. 2011) advised 7‐ to 14‐day antimicrobial courses but more recent studies indicate that the use of short duration therapy, as used in humans, may be as effective although different dosing regimes and antimicrobials still need to be more specifically evaluated (Weese *et al*. 2019). Empirical antibiotic choices should take into consideration the local susceptibilities of the uropathogens most likely to be implicated. It is, therefore, important to monitor changes in the aetiology and antibiotic resistance of UTIs in cats and dogs.

We detected bacteriuria in 30% (1547/5170) of the urinary samples submitted to the laboratories which was similar to previous reports (Guardabassi *et al*. 2015, KuKanich & Lubbers 2015). A low prevalence of samples containing two or more isolates was detected as most samples yielded the pure growth of a single organism; this is in agreement with previous studies (Ling *et al*. 2001, Ball *et al*. 2008, Lund *et al*. 2015, Rampacci *et al*. 2018). The number of urine samples from canine patients submitted for microbiological examination during the span of our study was double that of feline patients which may reflect that UTIs are more common in dogs (Buffington *et al*. 1997). Another possibility is that sampling is performed more readily in dogs than cats or the higher number of pet dogs than cats in the UK (26% and 31% of the UK households own cats and dogs) could have contributed to this difference (Murray *et al*. 2010). Positive samples were more commonly from older female dogs, which are known to be at greater risk of developing UTI (Bush 1976, Kivisto *et al*. 1977, Thomsen *et al*. 1986, Ling *et al*. 2001). The mean age of patients with significant bacteriuria, regardless of sex, was approximately 7 years in dogs and 11 years in cats, in agreement with previous studies (Lekcharoensuk *et al*. 2001, Ling *et al*. 2001, Cohn *et al*. 2003).

Amoxicillin, amoxicillin‐clavulanic acid, cephalexin, doxycycline and trimethoprim‐sulfamethoxazole are defined as oral antibiotics commonly used in the treatment of uncomplicated UTIs (sporadic UTIs in an otherwise healthy animal with a normal urinary tract anatomy and function) (Norris *et al*. 2000, De Briyne *et al*. 2014, BSAVA 2018). Fluoroquinolones and second or third‐generation cephalosporins are considered second‐line antibiotics and are to be used only where there is culture evidence that first‐line drugs will not be effective (Norris *et al*. 2000, Olin & Bartges 2015).

*E. coli* has been recognised as the most common bacterial cause of UTIs in dogs and cats as noted in our study (Ling *et al*. 2001, Penna *et al*. 2010, Hall *et al*. 2013, Dorsch *et al*. 2015, Moyaert *et al*. 2017). In agreement with similar studies, resistance above 20% was detected to cephalosporins by *E. coli* in our study, with the highest (64.4%) being against a first‐generation cephalosporin (cephalothin) and 31.2% resistant to a third‐generation cephalosporin (cefovecin) in canine *E. coli* (Weese *et al*. 2011, Rampacci *et al*. 2018). This indicates the need to continue monitoring resistance against cephalosporins, especially those of the third generation (*e.g*. cefovecin) and reserving them solely for difficult cases where culture and susceptibility indicate first‐line drugs are not susceptible in order to preserve their efficacy for human and veterinary medicine.

Overall, we observed a predominance of Gram‐negative species over Gram‐positive species, even though this difference was less marked in samples from feline patients due to a higher prevalence of *Enterococcus* spp. and coagulase‐negative staphylococci. Similarly to previous reports (Litster *et al*. 2007), *Enterococcus* spp. was the second most frequent urinary pathogen in cat samples. In humans, this opportunistic organism causes difficult‐to‐treat infections that usually affect geriatric patients with concurrent diseases and is often associated with implanted medical devices. Furthermore, intrinsic resistance of certain *Enterococcus* spp. such as *Enterococcus faecalis* to streptogramins and *Enterococcus faecium* to β‐lactam antibiotics have been previous reported (Fontana *et al*. 1996, Jackson *et al*. 2009). In our study, 60% of enterococcal isolates were from samples from patients above the age of 9 years. We detected resistance to erythromycin and tetracycline in approximately 90% and 50% of enterococcal isolates respectively from dogs and cats and although licensed, these antimicrobials are not recommended for UTIs in dogs and cats (BSAVA 2018, Weese *et al*. 2019). In addition, in vitro resistance does not necessarily correlate with bacteriological cure as most in vitro susceptibility breakpoints are based on achievable serum concentrations, which can be significantly lower than those attained in urine for antibiotics with renal excretion (such as amoxicillin) and in animals that can concentrate urine.

In our study, almost 10% of samples from cats with significant bacteriuria yielded the pure growth of coagulase‐negative staphylococci. The role of these organisms in canine and feline UTIs is not wholly understood but our findings give support to the view that they are relevant aetiologic agents of these infections. A previous study of urine samples from dogs with UTI implicated *Staphylococcus epidermidis*, *Staphylococcus simulans*, *Staphylococcus schleiferi* and *Staphylococcus saprophyticus* (Penna *et al*. 2010) and a more recent study has elucidated the species of coagulase‐negative staphylococci recovered from the urinary tracts of both cats and dogs (Moyaert *et al*. 2017). SIG including the major representative *Staphylococcus pseudintermedius* are the leading cause of staphylococcal infections in dogs, including UTIs (Lilenbaum *et al*. 2000, Ganière *et al*. 2005). It was the most frequently isolated coagulase‐positive staphylococci in our study and represented 4.4% and 7.0% of isolates from cat and dog samples, respectively.

Amoxicillin is recommended as a first‐line choice for the treatment of uncomplicated UTI (Weese *et al*. 2019). In our study, ampicillin was used to predict susceptibilities to amoxicillin, the latter is preferred for treatment as it has a better oral bioavailability. A considerable number of isolates, particularly *E. coli*, *S. pseudintermedius* and coagulase‐negative staphylococci, obtained from samples with significant bacteriuria were refractory to ampicillin. Overall, 36.9% of *E. coli* isolates from canine samples and 39.4% from feline samples were resistant to ampicillin. It is usually recommended that an antibiotic should not be used in the empirical treatment of UTIs if resistance rates exceed 10% to 20% among the most common uropathogens due to an increased risk of poor patient outcomes (Steen 2011). Our results suggest that ampicillin might not be a good antibiotic choice for the empirical treatment of canine and feline UTIs in the UK; however, they should be interpreted with caution. Studies can overestimate the prevalence of antibiotic resistance because they are often biased towards more serious cases (*e.g*. recurrent UTIs, patients that do not respond to empirical therapy) which are also more likely to be antibiotic resistant (Steen 2011). A lower prevalence of *E. coli* resistance to ampicillin (approximately 20%) was observed in a recent pan‐European study of UTI isolates from cats and dogs not recently exposed to antibiotics (Moyaert *et al*. 2017).

In our study, clavulanate potentiated amoxicillin was active in vitro against approximately 50% of isolates that were resistant to ampicillin alone. We detected 16.8% to 19.2% prevalence of amoxicillin‐clavulanic acid resistance among *E. coli* isolates, in agreement with previous reports (Marques *et al*. 2016). Our findings point to the need to monitor the levels of amoxicillin/amoxicillin‐clavulanic acid resistance in UTIs of cats and dogs. More recent studies indicate that there is limited evidence for the need to use clavulanic acid as an addition to amoxicillin and its use may not be essential, even with beta‐lactamase‐producing bacteria, due to the high concentrations of amoxicillin reached in urine (Weese *et al*. 2019). Trimethoprim‐sulfonamides are another advocated first‐line option for the empirical treatment of uncomplicated UTIs in dogs and cats (Weese *et al*. 2011, Rampacci *et al*. 2018) and, according to our results, might be superior to amoxicillin due to lower resistance rates. Although this should be interpreted with caution due to our low cat sample size (171), this antibiotic has had controversial activity against this bacterial species in other studies with relatively greater associated side effects (Weese *et al*. 2019).

It is generally recommended that, in order to preserve their effectiveness, the use of broad‐spectrum antibiotics should be reserved for cases that do not respond to narrow‐spectrum antibiotics and guided by documented in vitro susceptibility of the offending organisms (Weese *et al*. 2011). Enrofloxacin and marbofloxacin were the antibiotics routinely tested that showed greater in vitro activity against the most common uropathogens (overall susceptibility above 90%) despite previous reports showing an increase in resistance to fluoroquinolones (Cooke *et al*. 2002, Cohn *et al*. 2003) and reports after the conduction of this study showing similar results (Maluping *et al*. 2014, Marques *et al*. 2016, Rampacci *et al*. 2018). Research has been pointing to the efficacy of a high‐dose, short‐course regimen of enrofloxacin in attaining bacteriological cure in uncomplicated UTI cases in dogs and similar results have been obtained with trimethoprim‐sulfamethoxazole (Westropp *et al*. 2012, Olin & Bartges 2015). Short courses of antibiotics have the advantages of increasing owner compliance and reducing the costs and toxicity associated with treatment. Although fluoroquinolones can be effective in the treatment of UTIs, their use in animals is discouraged due to public health and antimicrobial resistance concerns (Weese *et al*. 2019).

In this study, we used the methods that were the standard of practice at the time of the study, including identification methods and CLSI breakpoints due to their specificity for bacteria isolated from animals (CLSI 2008). Since then new standards have been developed (CLSI VET01S 2020) which raise laboratory testing quality and should be used for previous and current studies in order to obtain the correct susceptibility data in all studies, but this process is far from complete. For example, there are far more breakpoints for pathogens from dogs than those from cats and we lack breakpoints on certain important bacteria which highlights the need to continuously update these standards (Moyaert *et al*. 2017). In addition, the fact that information on the method of urine collection was often not provided by veterinary surgeons may have further impacted the results as different cut‐off for significance are used depending on the collection method. The cut‐off for this study was ≥10^5^ CFU/mL but significant pathological infections in cystocentesis samples may not have been included as they can be detected at <10^5^ and >10^3^ CFU/mL (Sørensen *et al*. 2016).

*E. coli* continues to be the most common cause of UTIs in dogs and cats with the detection of notable resistance to cephalosporins in our study. *Enterococcus* spp. were frequently detected in cat samples with high resistance to erythromycin and tetracycline and low resistance to ampicillin and amoxicillin‐clavulanic acid. Our findings support the view that *Staphylococcus* spp. are relevant aetiologic agents of bacterial UTIs indicating the need for further studies on the species of coagulase‐negative staphylococci associated with these infections. Considerable resistance to ampicillin and amoxicillin‐clavulanic acid from several isolates was identified which is in contrast to that of trimethoprim‐sulfamethoxazole which was low. Enrofloxacin and marbofloxacin showed the best in vitro activity against the isolates tested. Our findings provide evidence of the emergence of resistance to antibiotics commonly used in the treatment of UTIs in dogs and cats among the uropathogens most likely to be responsible for these infections in the UK. Changes in the aetiology and antibiotic resistance of UTIs should be continuously monitored to evaluate the need to introduce changes in the recommendations (*e.g*. ISCAID guidelines, Weese *et al*. 2019) about empiric therapy when antimicrobial susceptibility testing is not possible or pending.

### Conflict of interest

None of the authors of this article has a financial or personal relationship with other people or organisations that could inappropriately influence or bias the content of the paper.

### Ethical statement

This work would be likely to gain approval in Europe under the European Directive 2010/63/EU (on the protection of animals used for scientific purposes) or an appropriate clinical ethics committee.
